# Exploring the Relationship Between Senescence and Colorectal Cancer in Prognosis, Immunity, and Treatment

**DOI:** 10.3389/fgene.2022.930248

**Published:** 2022-06-15

**Authors:** Kechen Dong, Jianping Liu, Wei Zhou, Guanglin Zhang

**Affiliations:** ^1^ Department of Oncology of Head and Neck, Huangshi Central Hospital (Pu Ai Hospital), Affiliated Hospital of Hubei Polytechnic University, Edong Healthcare Group, Huangshi, China; ^2^ Department of Abdominal and Pelvic Medical Oncology II Ward, Huangshi Central Hospital (Pu Ai Hospital), Affiliated Hospital of Hubei Polytechnic University, Edong Healthcare Group, Huangshi, China; ^3^ Department of Urology, Huangshi Central Hospital (Pu Ai Hospital), Affiliated Hospital of Hubei Polytechnic University, Edong Healthcare Group, Huangshi, China

**Keywords:** senescence, colorectal cancer, prognostic model, tumor immune microenvironment, immunothearpy

## Abstract

**Background:** Senescence, as an effective barrier against tumorigenesis, plays a critical role in cancer therapy. However, the role of senescence in colorectal cancer (CRC) has not yet been reported. This study aimed to build a prognostic signature for the prognosis of patients with CRC based on senescence-related genes.

**Methods:** A prognostic signature was built from TCGA based on differentially expressed senescence-related genes by the least absolute shrinkage and selection operator (LASSO) and Cox regression analyses, which were further validated using two Gene Expression Omnibus (GEO) cohorts. The CIBERSORT and ssGSEA algorithms were utilized to analyze the infiltrating abundance of immune cells. The relationship of signature with the immune therapy and the sensitivity of different therapies was explored.

**Results:** We found 93 genes associated with senescence that were differentially expressed. Based on expression and clinical parameters, we developed a senescence-related prognostic signature and its effectiveness was verified using two external validation cohorts. Overall survival was predicted using a prognostic nomogram that incorporated the predictive values of the risk score and clinical traits. Additionally, the risk score was significantly correlated with immune cells infiltration, tumor immune microenvironment (TME) score, immune checkpoints, immunotherapeutic efficacy, and chemotherapy sensitivity.

**Conclusion:** The senescence-related prognostic model can well predict the prognosis, immunotherapeutic response, and identify potential drug targets, which can help guide individualized treatment.

## Introduction

Colorectal cancer (CRC) is one of the most common malignant tumors. Its incidence rate ranks third in the world and the mortality rate is ranked second (Sung et al., 2021). There were nearly 1.9 million (10.0%) new cases of CRC worldwide, followed by breast and lung cancer in incidence (Sung et al., 2021). CRC is a malignant tumor of the digestive system and is the first tumor in the world in terms of morbidity and mortality and seriously threatens the life and health of individuals (Siegel et al., 2020). At present, the main treatment methods for CRC include a combination of endoscopic resection, surgical resection, chemotherapy, and radiotherapy (Modest et al., 2019; Dariya et al., 2020). As the surgical intervention is available for early CRC, a large majority of patients with advanced CRC suffer from a poor therapeutic outcome with higher rates of malignant recurrence and distant metastases, resulting in a 5-years survival rate of less than 10% (Chen et al., 2021a). Hence, it is particularly important to find a prognostic model that can accurately classify CRC patients, so that appropriate treatment method can be selected for patients with different precise prognoses.

Cellular senescence is defined as a permanent state of cell cycle termination. It is a response to endogenous and exogenous stresses, including DNA damage, telomere dysfunction, oncogene activation, and organelle stress, and is associated with processes such as tumor suppression, tissue repair, embryogenesis, and organ aging (López-Otín et al., 2013; Di Micco et al., 2021). The current state of aging research shares many similarities with cancer research over the past few decades. In the newly proposed third edition of cancer hallmarks in 2022, four new members have been added, and one of the hallmarks is senescent cells (Hanahan, 2022). Cancer is the result of abnormally enhanced cellular fitness, whereas senescence is characterized by loss of fitness. On the surface, cancer and aging appear to be opposite processes. However, at a deeper level, the two may have a common origin. Cellular senescence is caused by a time-dependent accumulation of cellular damage (Gems and Partridge, 2013). Meanwhile, cell damage occasionally confers abnormal benefits on certain cells, ultimately leading to cancer. Therefore, cancer and aging can be thought of as two distinct manifestations of the same underlying process, the accumulation of cellular damage. Numerous genes have been implicated in cellular senescence as biomarkers and causal drivers (Giovannini et al., 2012; Jia et al., 2018; Li et al., 2019; Shaosheng et al., 2021). Li et al. (Li et al., 2019) found that knockdown of BAZ1A-KD results in up-regulation of SMAD3 expression, which in turn activates transcription of the p21-encoding gene CDKN1A and causes senescence-related phenotypes in human cancer cells. However, it is unknown if these senescence-related genes have an impact on CRC prognosis.

In this study, for the first time, we established a prognostic signature based on differentially expressed senescence-related genes (DEGs) and verified its accuracy in two external databases. Following that, we developed a nomogram to predict the OS of patients with CRC. In addition, we investigated the prognostic value, and impact on tumor immune infiltration, immune checkpoint expression, immunotherapy, and chemotherapeutic drug sensitivity of senescence-related genes in HCC.

## Materials and Methods

### Data Source

A total of 279 senescence-related genes were collected from CellAge database (https://genomics.senescence.info/cells/signatures.php?) and were listed in Supplementary Table S1. The RNA-seq expression and clinical traits for CRC patients were obtained and extracted from three independent CRC cohorts (TCGA-COD, n = 477; GSE39582, n = 556; GSE17536, n = 175). IMvigor210 with immunotherapy data and clinical information were obtained from the IMvigor210CoreBiologies R package. TCGA cohort was used to build the signature, and two GEO cohorts were used to externally verify the signature.

### Establishment and Identification of Prognostic Signature

The training cohort was employed to detect the senescence-related DEGs between normal and CRC tissues via the R package “limma” in RStudio, with the following cutoff for adjustment: *p*-value < 0.05 and |fold change (FC)| > 1.5. To screen senescence-related genes with prognostic significance, a univariate Cox regression analysis was conducted on DEGs. Following that, the Least absolute shrinkage and selection operator (LASSO) and multivariable Cox analysis was performed to build a predictive signature. The following formula was employed to calculate the risk scores of CRC samples:

$$Rish\;Score=\sum_{i=1}^{n}coef\left(Xi\right)\;\times \;\exp\;\left(Xi\right)$$

“Coef”, “exp”, and “n” represented the coefficient of the gene, the expression level, and the number of genes, respectively. The median risk score was used as the threshold. Patients with risk scores greater than the threshold (median risk score) were included in the high-risk group and the rest in the low-risk group. Receiver operating characteristics (ROC) and Kaplan-Meier survival curves were employed to assess the effectiveness of the signature. Principal component analysis (PCA) was conducted to verify whether the risk score could distinguish high- and low-risk score groups.

Two GEO validation cohorts were recruited to vertify the predictive accuracy of the model developed from the TCGA set. The above cut-off value was used to divide all CRC patients into high- and low-risk score groups, the same method was employed for the predictive power of the signature in OS prediction.

### Nomogram Construction and Assessment

We explored the risk score with the corresponding CRC samples’ clinical information, including age, gender, tumor site, and TNM stage. Additionally, we also explored whether the risk levels would affect the prognosis of patients in distinct clinical variable groups. Univariate and multivariate models were employed to ascertain whether the signature could be an independent predictive indicator for the prognosis of CRC patients. Then, a nomogram integrating risk score and clinical parameters was built using the “rms” R packages. ROC and calibration curves were employed to validate its accuracy is demonstrated.

### Immune Activities Analysis

The ssGSEA algorithm was used to quantify the scores of 16 tumor immune infiltration cells (TIICs) and the function of 13 immune-related pathways. The proportion of 22 TIICs in two risk score groups was further quantified with CIBERSORT algorithm. The immune score, stromal score, ESTIMATE score were calculated through ESTIMATE algorithm to quantify the tumors microenvironment (Arbour et al., 2021). Two immune checkpoints (PD-1 and PD-L1) were chosen to assess the differences in their expression levels in two risk subgroups.

### Targeted Drug and Immunotherapy

In this study, the capability of the signature in predicting sensitivity of chemotherapy and immunotherapy was investigated. In the aspect of chemotherapy, half maximal inhibitory concentration (IC50) was used to predict the sensitivity of chemotherapy drugs in the high- and low-risk groups. Meanwhile, potential immune checkpoint inhibitors (ICIs) response was predicted with TIDE algorithm (Jiang et al., 2018).

### Gene Set Enrichment Analysis

To investigate the biological pathways of the subgroups, we further generated a gene set enrichment analysis (GSEA) for functional enrichment analysis. Gene sets with *p*-value and Q-value < 0.05 were the cutoff criterion for significant gene enrichment.

### Statistical Analysis

Data were analyzed by R software version 4.1.0. Log-rank test was used for survival analysis. Wilcoxon rank-sum or Kruskal–Wallis tests were utilized to compare differences between two or three groups, respectively. The ROC curves were plotted to access the prognostic value of the model.

## Results

### PPI Network and GO and KEGG Enrichment Analyses

**FIGURE 1:**
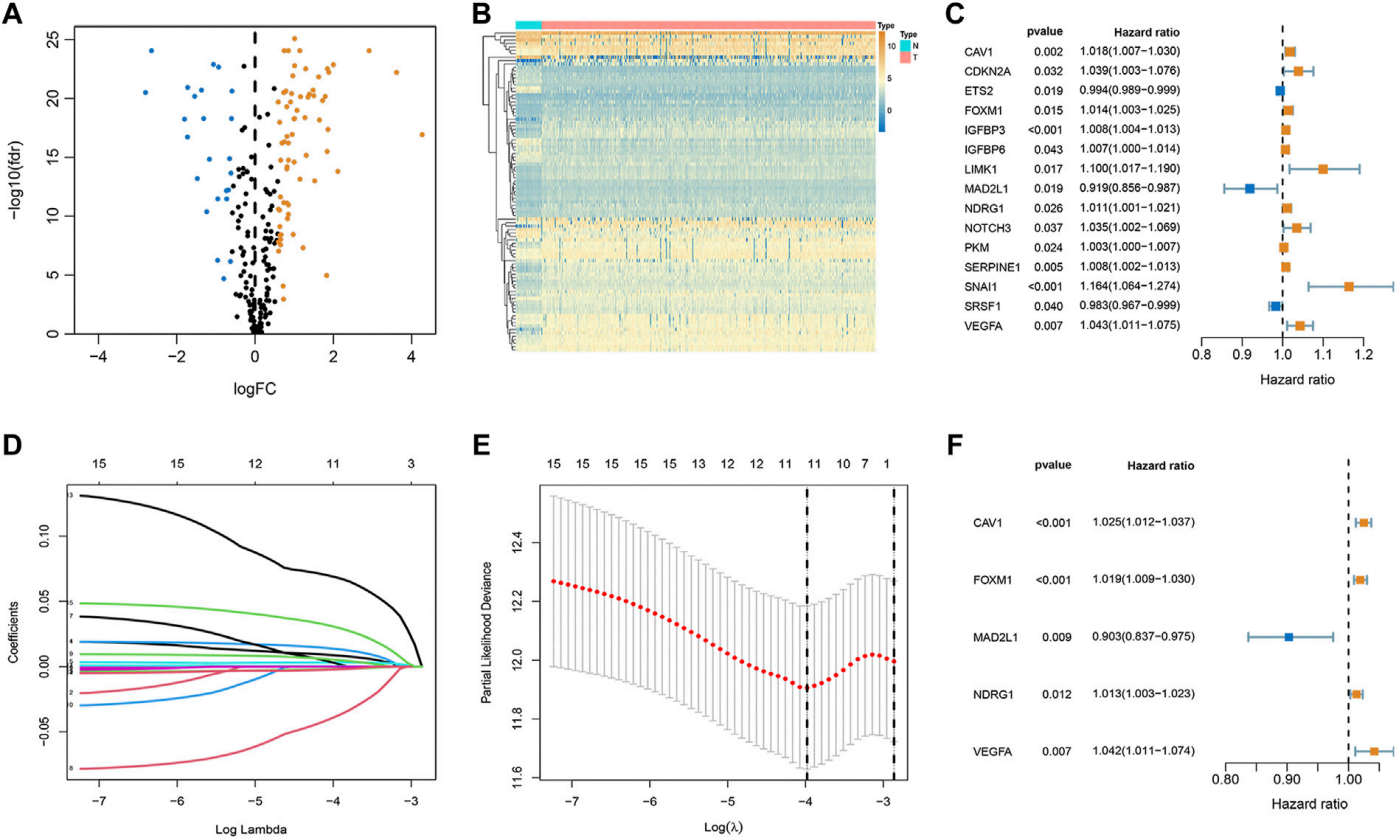
Establishment of optimal senescence-related signature in the TCGA set. **(A,B)** Volcano and heatmap representations of lactate-related DEGs between normal and CRC groups. **(C)** The prognostic genes were selected by univariate Cox regression analysis. **(D,E)** Lasso regression analysis. **(F)** Multivariate Cox regression analyses of the association between genes and OS of patients.

Among 279 senescence-related genes, 93 DEGs in CRC patients of the TCGA cohort were identified with FDR <0.05 and FC > 1.5. Volcano and heatmap representations of senescence-related DEGs are provided in Figures 1A,B. Then, Protein-protein interaction (PPI) networks and functional enrichment analyses were constructed to comprehensively investigate these DEGs. As shown by PPI analysis, 84 of these 93 DEGs formed interaction modules (Supplementary Figure S1A). By using the cytoHubba plugin, we screened 10 hubgenes (Supplementary Figure S1B). GO functional annotation showed that the 93 DEGs are mainly related to regulation of cell cycle phase transition, transcription regulator complex, and DNA-binding transcription factor binding (Supplementary Figure S1C). KEGG signaling enrichment annotation showed that these DEGs are mainly enriched in the cell cycle, cellular senescence, p53 signaling pathway, and other tumor-related signal pathways (Supplementary Figure S1D).

### Establishment and Validation of the Senescence-Related Signature

To explore whether these senescence-related genes are related to the prognosis of CRC, univariate COX regression analysis was applied. Based on the TCGA cohort, 15 genes were identified (Figure 1C). As shown in Figures 1D,E, 15 genes were subject to LASSO Cox regression analysis to avoid overfitting, and 11 out of 15 genes were chosen as the appropriate candidates for constructing a risk signature. Subsequently, multivariate Cox regression analysis obtained 5 genes (CAV1, FOXM1, MAD2L1, NDRG1, and VEGFA) to build a prognostic signature (Figure 1F). Risk score = expression (CAV1) × 0.024 + expression (FOXM1) × (0.019) + expression (MAD2L1) × (-0.102) + expression (NDRG1) × (0.012) + expression (VEGFA) × (0.041). Median risk scores divided the cohort of CRC patients into the low- and high-risk subgroups. To analyze the translational levels of the signature genes, the Human Protein Atlas (HPA) database can be used, showing the expression and localization of the corresponding protein. The results showed that FOXM1, MAD2L1, NDRG1, and VEGFA was highly expressed in CRC tissue, wihle CAV1 was lowly expressed in CRC tissue (Supplementary Figure S2).

### Internal and External Validation of the Signature

**FIGURE 2:**
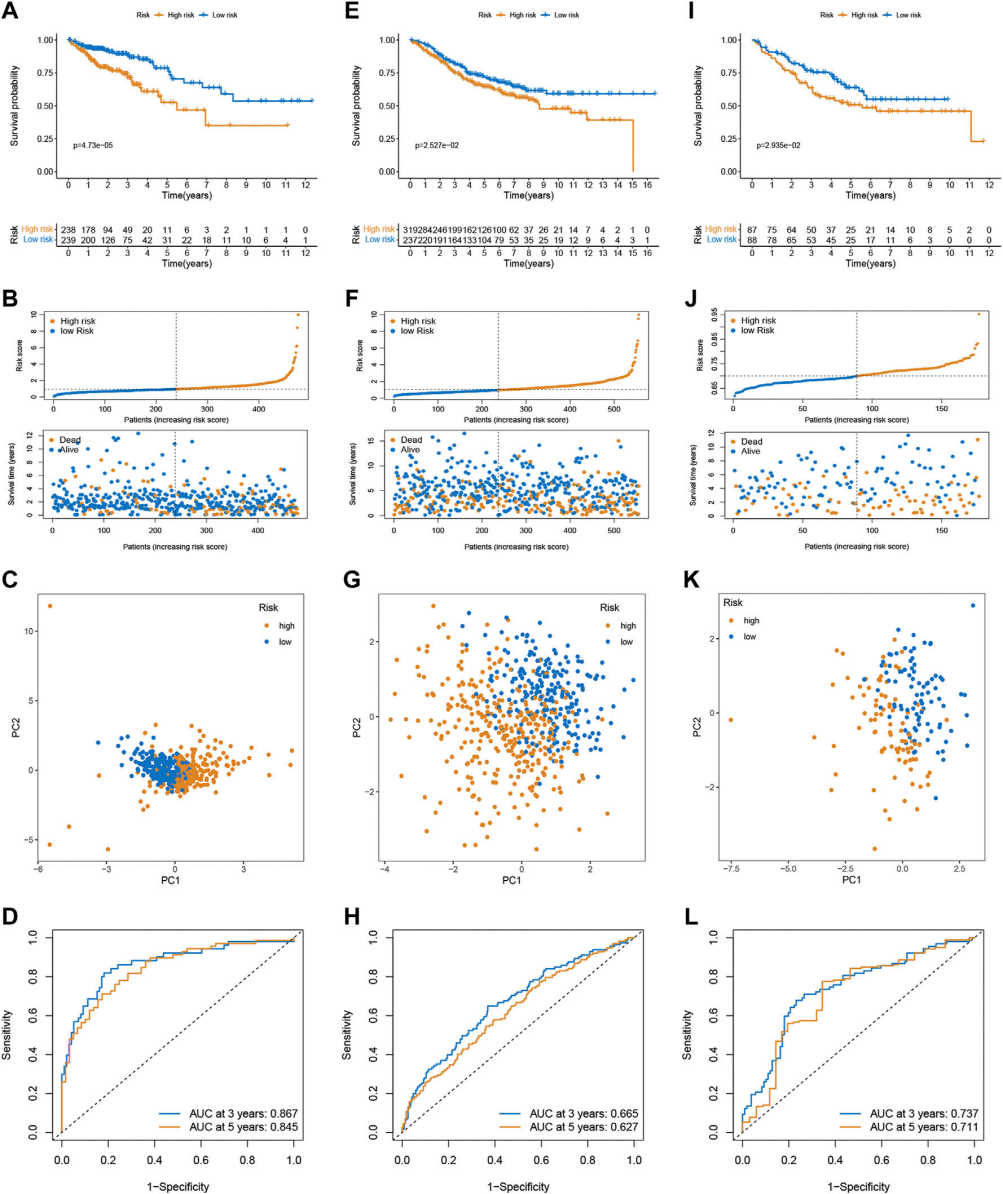
Validation of the prognostic prediction performance of the signature. Kaplan-Meier survival analysis in the training **(A)**, GSE39582 **(E)**, and GSE17536 cohort **(I)**. Distribution of risk score and survival status in the training **(B)**, GSE39582 **(F)**, and GSE17536 cohort **(J)**. PCA analysis in the training **(C)**, GSE39582 **(G)**, and GSE17536 cohort **(K)**. Time-dependent ROC curves of risk scores in the training **(D)**, GSE39582 **(H)**, and GSE17536 cohort **(L)**.

In the training cohort, the Kaplan-Meier analysis revealed that high-risk group had lower OS compared to low-risk group (*p* < 0.001, Figure 2A). Also, mortality was increased in CRC patients with increasing risk scores (Figure 2B). PCA analysis revealed that there was a clear division in two risk subgroups (Figure 2C). ROC plots were also used to assess diagnostic efficiency, with AUCs of 0.867 and 0.845 for 3 and 5-years survival, respectively (Figure 2D).

To confirm the robustness of the signature, the risk scores of CRC patients were calculated in two external validation sets (GSE39582 and GSE17536) using the same formula, and divided patients into the high- and low-risk subgroups according to the cutoff of the training cohort. Likewise, high-risk was associated with OS (Figures 2E,I), and the number of deaths increased with increasing risk scores (Figures 2F,J). PCA demonstrated overt separation of both subgroups (Figures 2G,K). The ROC further indicated the predicting accuracy of the signature (Figures 2H, 2L). Additionally, the Imvigor210 dataset of the treatment response data of patients who underwent anti-PD-L1 immunotherapy was retrieved to validate the predictive ability of the senescence-based signature in ICI therapy. Kaplan-Meier analysis showed that a high-risk score was associated with a poorer survival rate than a low risk score (Supplementary Figure S3).

### Prognostic Value of the Signature

**FIGURE 3:**
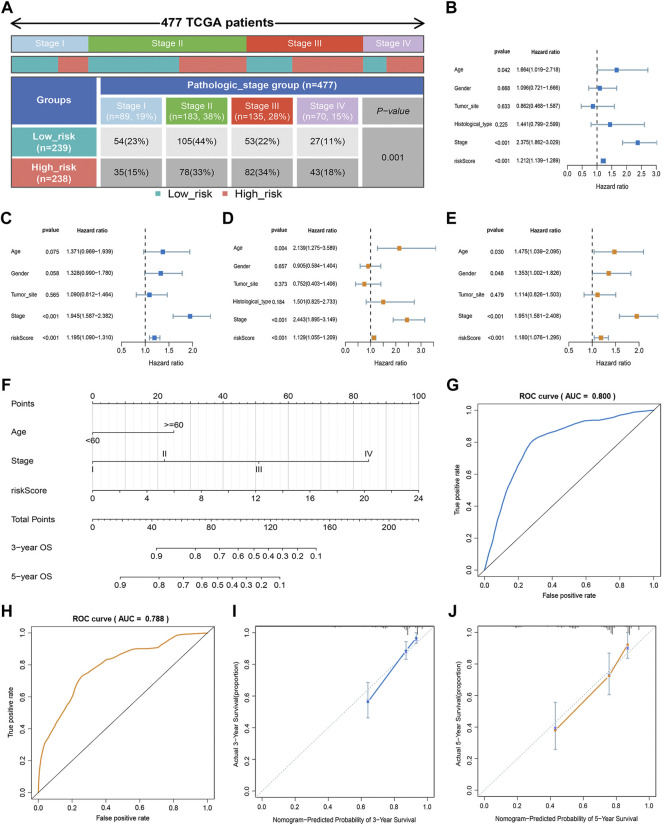
Clinical value of the risk score. **(A)** Association of risk score with TNM stage. **(B,C)** Univariate analysis of risk scores and clinicopathological parameters in the training **(B)** and GSE39582 cohort **(C)**. **(D,E)** Multivariate Cox regression analysis of risk scores and clinicopathological parameters in the training **(D)** and GSE39582 cohort **(E)**. **(F)** Prediction of the nomogram based on clinical traits and risk score. **(G,H)** ROC curves of the nomogram for OS prediction at three **(G)** and 5 years **(H)**. **(I,J)** Calibration curve of the nomogram for predicting OS rates at three **(I)** and 5 years **(J)**.

In our study, we analyzed the prognosis of patients in low- and high-risk groups among distinct clinical variable subgroups. As shown in Supplementary Figure S4, patients with high-risk scores had poorer survival probabilities than those with low-risk scores in all distinct clinical variable subgroups. In addition, we further investigated the association between risk scores and each clinical characteristic. The results showed that the risk score was linked to the TNM stage (*p* < 0.01; Figure 3A). Subsequently, we verified the independence and applicability of the risk score in the training and GSE39582 sets. Univariate and multivariate Cox regression analysis results showed that the signature could independently predict the prognosis of CRC patients, regardless of age, gender, tumor site, histological type, and TNM stage (*p* < 0.001, Figures 3B–E).

### Development and Assessment of the Nomogram

An approach by which 3- and 5-years OS rates could be more accurately predicted was to construct a nomogram model based on Cox regression results (Figure 3F), which included risk score, age, and TNM stage. As shown in Figure 3F, this nomogram can predict the 3- and 5-survival for a patient based on the sum of the scores. The ROC curve revealed the high accuracy of the nomogram for 3-years (AUC = 0.80) and 5 -year (AUC = 0.788) survival rates (Figures 3G,H). The calibration curves comparing the predicted and actual survival rates of CRC patients indicated that the predicted survival rates were in good agreement with those actual rates (Figures 3I,J).

### Correlations Between the Risk Scores and TME

**FIGURE 4:**
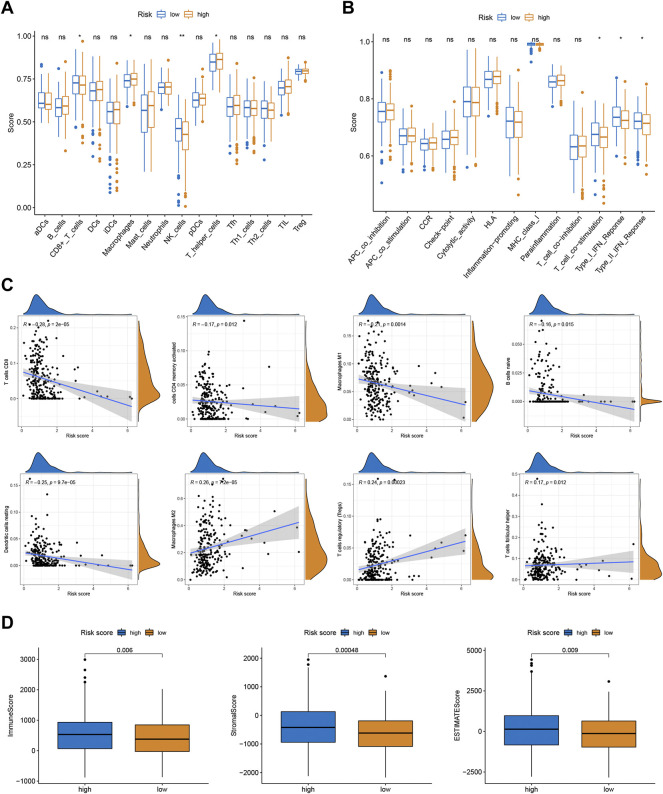
Correlations between the risk scores and TME. **(A)** The ssGSEA scores of immune infiltrating cells. **(B)** The ssGSEA scores of immune functions. **(C)** The proportion of 22 immune infiltrating cells in two risk subgroups. **(D)** TME score in two risk subgroups.

To better investigate the relationship between risk score and immune characteristics, ssGSEA was used to calculate the enrichment scores of various immune cells. According to Figure 4A, the relative scale of fraction for CD8^+^ T cells and NK cells was obviously lower in the high-risk group than that in the low-risk group. On the contrary, the fraction of macrophages and T helper cells were much lower in the low-risk group. We also found substantial variations in immune function in terms of T cell co-stimulation, type I IFN response, and type II IFN response (Figure 4B). Furthermore, CIBERSORT algorithms were employed to calculate the scores of various TIICs. Results suggested that the infiltration abundance of CD8^+^ T cells, memory activated CD4^+^ T cells, macrophages M1, naive B cells, and resting dendritic cells in the high-risk group was obviously lower than that in the low-risk group, and their infiltration abundance decreased with increasing risk score (Figure 4C). However, the infiltrative abundance of M2 macrophages, T cells regulatory (Tregs), and Tfh cells was distinctly higher in the high-risk group, and their abundance increased prominently with risk scores increased (Figure 4C). In addition, patients with a low-risk score presented a higher level of the immune score, stromal score, and ESTIMATE score than those with a high-risk score (Figure 4D).

### Relationship Between the Signature and CRC Therapy

**FIGURE 5:**
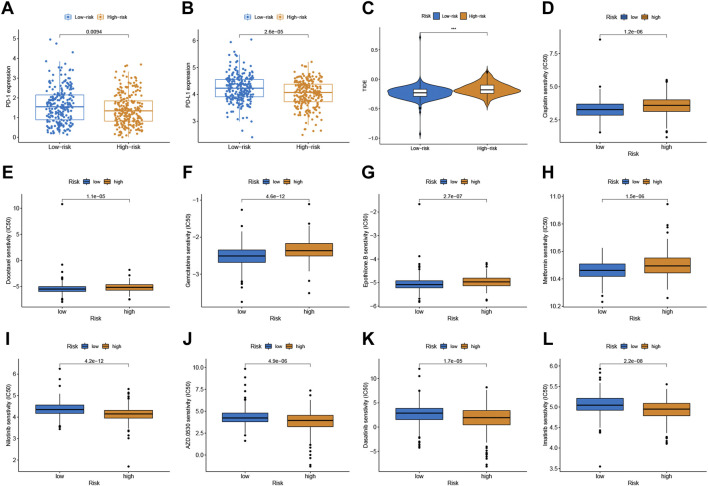
Correlation between the predictive signature and CRC therapy. **(A,B)** The expression value of PD-1 and PD-L1 between two risk subgroups. **(C)** Comparison of TIDE score between low- and high-risk subgroups. **(D–L)** Estimated drug sensitivity in patients with high- and low-risk subgroups.

Given the significance of checkpoint treatment, we investigate more into the variations in immune checkpoint expression between different risk subgroups. The results indicated that the expression of PD-1 and PD-L1 in the low-risk group were higher than those in the low-risk group (Figures 5A,B). Furthermore, we applied the TIDE algorithms to evaluate the effectiveness of the signatures in forecasting ICIs responsiveness in CRC. TIDE scores were higher in the high-risk score group compared to the low-risk group (Figure 5C). Taken together, the signature can predict the benefit of CRC immunotherapy.

Chemotherapeutic drug sensitivity analysis will help guide the optimal selection of commonly used chemotherapeutic drugs for CRC. By comparing IC50 values in high- and low-risk groups, Wilcoxon signed-rank test was used to evaluate chemosensitivity. The result indicated that the patients with low-risk scores were more sensitive to cisplatin, docetaxel, gemcitabine, epothilone B, and Metformin, while patients with the high-risk score were more sensitive to nilotinib, saracatinib (AZD0530), dasatinib, and imatinib (Figure 5D-L).

### GSEA Enrichment Analysis

**FIGURE 6:**
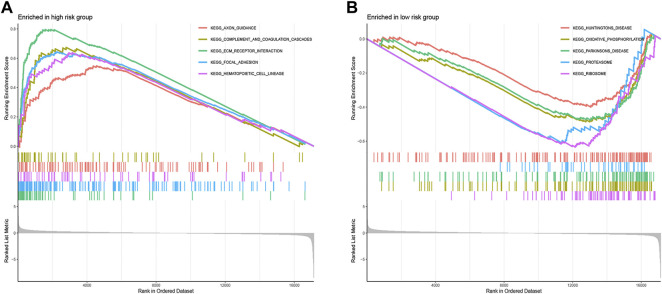
Functional enrichment analysis between low- and high-risk groups. The top five signaling pathways in the high- **(A)** and low-risk subgroup **(B)**.

To clarify the important pathway of signature enrichment related to pyroptosis, we conducted GSEA. As shown in Supplementary Table S2, 55 enrichment pathways with significant variations between low and high-risk groups were identified at the criteria of FDR <0.25, *p* < 0.05. The top five signaling pathways in the high-risk group were axon guidance, complement and coagulation cascades, ECM receptor interaction, focal adhesion, and hematopoietic cell lineage (Figure 6A). The top five signaling pathways in the low-risk group were huntingtons disease, oxidative phosphorylation, parkinsons disease, proteasome, and ribosome (Figure 6B).

## Discussion

CRC is a highly heterogeneous disease, and survival time varies widely among patients with similar clinical stages. Cellular senescence is recognized as an effective barrier against tumorigenesis and can be promoted by immune surveillance (Ou et al., 2021). Most research on cellular senescence has focused on non-tumor cells, but tumor cells can also undergo senescence. The treatment of cancer consisting of pro-senescence and senolytic therapy has also been explored, which is expected to become new approaches for targeted therapy of cancer (Wang et al., 2022). Increasing evidence suggests that senescent cells can be eliminated by senescence-associated secretory phenotype (SASP)-elicited immune responses involving both innate and adaptive immunity, so activation of the host immune system is a particularly attractive approach to clearing senescent cancer cells (Schneider et al., 2021; Wang et al., 2022). However, the correlation between cellular senescence and TME remains unclear, and the value of cellular senescence-related genes in assessing immune infiltration and clinical outcome in CRC has not been reported. Therefore, this study aimed to establish a new prognostic signature based on senescence-related genes to help accurately predict the prognosis of CRC patients and guide individualized treatment.

In this work, we analyzed the role of senescence in CRC using the public databases. And 93 differentially expressed senescence-related genes were identified between CRC and normal samples. To comprehensively explore the mechanism of senescence in CRC, we performed univariate Cox regression analysis and LASSO Cox regression analysis on these DEGs to develop a senescence-related signature in the training cohort. The signature contained five senescence-associated genes: CAV1, FOXM1, MAD2L1, NDRG1, and VEGFA. CAV1 (caveolin-1) is a key structural component of caveolae and plays an important role in a variety of cellular processes including cholesterol homeostasis, vesicle transport, and tumor progression (Ha and Chi, 2012). CAV1 has been shown to play a dual role in tumorigenesis, inhibiting or promoting tumor growth depending on the cellular context (Ha and Chi, 2012; Kamposioras et al., 20221080). Several studies have reported the effect of CAV1 expression on CRC, but there were no consistent results (Alshenawy and Ali, 2013; Xue et al., 2015; Zhao et al., 2015). Typically, CAV1 expression is elevated in CRC tissue compared to adjacent normal tissue (Alshenawy and Ali, 2013; Xue et al., 2015). CAV1 expression was associated with clinicopathological traits and prognosis of CRC patients (Xue et al., 2015; Yang et al., 2018). CAV1 can affect the occurrence and development of CRC through different mechanisms, including via activation of SLC2A3/GLUT3 transcription (Ha and Chi, 2012), suppressing phosphorylation of epidermal growth factor receptor (Yang et al., 2018), and stimulating HMGA1-mediated GLUT3 transcription (Ha et al., 2012). FOXM1, a member of FOX superfamily, has been implicated in CRC progression and chemoresistance (Varghese et al., 2019; Yang et al., 2019; Yang et al., 2020). Yang et al. (Yang et al., 2019) revealed that FOXM1 expression significantly elavated in CRC tissues and was positively linked to tumor size, TNM stage, lymphatic and distant metastasis. Overexpression of FOXM1 promoted oncogenic effects on CRC by activating the β-catenin signaling pathway. Varghese et al. (Varghese et al., 2019) showed that FOXM1 regulates 5-FU resistance in CRC by regulating TYMS expression. Yang et al. (Yang et al., 2020) FOXM1 simultaneously promote migration, invasion, and drug resistance of CRC cells through upregulating Snail. MAD2L1, as a member of the spindle checkpoint functional complex, plays a crucial role in cell cycle regulation (Zhong et al., 2015). MAD2L1 has been reported as a novel oncogene that plays a role in regulating cancer cell growth and apoptosis (Ding et al., 2020; Ding et al., 2022). NDRG1 has been reported to act as a metastasis suppressor (Bae et al., 2013; Sahni et al., 2014). A recent study shows that NDRG1 regulates filopodia-induced CRC invasiveness by regulating CDC42 activity (Aikemu et al., 2021). VEGFA is an endothelial growth factor and regulator of vascular permeability (Claesson-Welsh and Welsh, 2013). Increasing evidence suggests that VEGFA-dependent signaling pathways play crucial roles in CRC progression (Terme et al., 2013; Dai et al., 2020; Liu et al., 2020).

Furthermore, all CRC patients were categorized into low- and high-risk subgroups depending upon the median value. Internal and external validation results showed that risk scores independently and effectively predicted 3- and 5-years survival in CRC patients. We also conducted univariate and multivariate Cox analyses to explore the effectiveness of the signature and clinical parameters as indicators of patient prognosis. It was concluded that the risk score served as an independent prognostic predictor for CRC patients. To better quantify the 3- and 5-years survival of CRC samples, a nomogram, combined with these independent indicators, was constructed. The predictive accuracy of the nomogram was verified by the ROC curve and calibration plot. Therefore, it may be used as a supplementary tool to better assist the prognosis evaluation and treatment of CRC.

We calculated the infiltration of immune cells and TME scores in the high-and low-risk groups. The ssGSEA and CIBERSORT results showed the risk score was closely related to the relative contents of TIICs, especially for T cells and macrophages. And with the increase of the risk score in the prognostic signature, relative contents of CD8^+^ T cells tended to be downregulated, while the relative contents of macrophages tended to be upregulated. This discovery is in line with prior research that intratumoral T cell density has been shown to be an independent prognostic factor in CRC (Galon et al., 2006; Miller et al., 2021). CD8^+^ T cells are considered major drivers of anti-tumor immunity (van der Leun et al., 2020). Accumulating evidence suggests that increased tumor-related macrophage infiltration results in a poor prognosis in CRC (Wei et al., 2019). Tumor-associated macrophage-induced immune responses were already considered critical determinants of tumor progression (Pan et al., 2020). Tumor-associated macrophages can also perform pre-tumor activities such as enhancing tumor cell proliferation, and invasion, angiogenesis, and inhibiting anti-tumor immune surveillance (Chen et al., 2021b; Boutilier and Elsawa, 2021). Also, patients with low risk score have a higher TME score than those with high risk score.

Emerging therapeutic strategies, including PD-1/PD-L1 inhibitors, are used for treating CRC (Yaghoubi et al., 2019). In our study, the expression levels of PD-1 and PD-L1 in the low-risk group were higher compared to those in the high-risk group, which implied that the signature would be able to predict their expression levels and provide guidance during immunotherapy with ICIs. Furthermore, we found that patients with high-risk scores had a higher TIDE score than those with the low-risk score. A lower TIDE score indicates a lower possibility of tumor immune evasion and may benefit from immunotherapy, which further explains the better prognosis of patients in the low-risk group in our study. These findings provide a basis for a more comprehensive understanding of anti-tumor immune responses in CRC patients, as well as guidance for personalized immunotherapy treatments. Chemotherapy and immunotherapy are the most important adjuvant therapies for CRC, which are of great significance for improving both the prognosis of patients and their quality of life. Patients with low risk score was more sensitive to cisplatin, docetaxel, gemcitabine, epothilone B, and Metformin, while patients with the high-risk score were more sensitive to nilotinib, saracatinib, dasatinib, and imatinib. The combination of chemotherapy and immunotherapy can provide precise and individualized therapy for patients with a different risk scores.

## Conclusion

This study successfully constructed a 5-gene senescence-related signature that could be used to classify CRC patients. The prognostic model shows the convincing clinical value and may provide new ideas for improving the OS rate of CRC patients and facilitating personalized treatment.

## Data Availability

Publicly available datasets were analyzed in this study. The TCGA and GEO databases can be found here: https://portal.gdc.cancer.gov/ and https://www.ncbi.nlm.nih.gov/geo/, respectively.
