# Supplementary material for: Exploring the Relationship Between Senescence and Colorectal Cancer in Prognosis, Immunity, and Treatment
Source: Front Genet. 2022 Jun 15;13:930248. doi: 10.3389/fgene.2022.930248 (PMC9240351; doi:10.3389/fgene.2022.930248)
Supplement: Supplementary file 2 [file Table1.DOCX]

Table S1 Summary of 279 recognized senescence-related genes

| ACLY | CSNK2A1 | IGFBP6 | MORF4 | CDK6 | HDAC4 | MAP4K1 | PLA2R1 |
| --- | --- | --- | --- | --- | --- | --- | --- |
| AAK1 | CXCL1 | IGFBP5 | MXD4 | CDK4 | HDAC1 | MAP3K7 | PKM |
| ABI3 | DDB2 | IL1A | MVK | CDKN1A | HEPACAM | MAPK12 | PML |
| ADCK5 | CYR61 | IL8 | MYC | CDKN1C | HJURP | MAPKAPK5 | PNPT1 |
| AKR1B1 | DEK | ING1 | MYLK | CDKN1B | HIVEP1 | MARCH5 | PMVK |
| AGT | DGCR8 | ING2 | NADK | CDKN2A | HK3 | MAPK14 | POT1 |
| AKT1 | DHCR24 | IRF3 | NANOG | CDKN2AIP | HMGB1 | MAST1 | POU5F1 |
| ALOX15B | DLX2 | IRF5 | NDRG1 | CDKN2B | HRAS | MATK | PPM1B |
| AR | DHX9 | IRF7 | NEK1 | CENPA | HSPA5 | MCL1 | PPM1D |
| ARPC1B | DPY30 | ITPK1 | NEK4 | CEBPB | HSPB2 | MDH1 | PRMT6 |
| ASF1A | DUSP3 | ITGB4 | NEK6 | CHEK1 | ID1 | MCRS1 | PRKCH |
| ASPH | DUSP16 | ITPKB | NFE2L2 | CKB | ID4 | MECP2 | PRKCD |
| ATF7IP | E2F1 | ITSN2 | NINJ1 | CPEB1 | IGFBP1 | MOB3A | PROX1 |
| ATM | EHF | KCNJ12 | NOTCH3 | CSNK1A1 | IFNG | MMP9 | PRPF19 |
| AURKA | ENDOG | KDM4A | NOX4 | CTNNAL1 | IGFBP3 | MORC3 | PSMB5 |
| AXL | EPHA3 | KDM5B | NR2E1 | PTRF | TERC | SREBF1 | SMARCB1 |
| BAG3 | ERRFI1 | KIAA1524 | NTN4 | PTTG1 | TBX2 | SRSF1 | SMURF2 |
| BHLHE40 | ETS1 | KL | NUAK1 | PSMD14 | TERF2 | STAT5B | SNAI1 |
| BCL6 | ETS2 | KSR2 | OTX2 | RAD21 | TERT | STK32C | SOCS1 |
| BLK | EWSR1 | LATS1 | P3H1 | RAF1 | TFAP4 | STK40 | SOD1 |
| BLVRA | FASTK | LEO1 | PATZ1 | RB1 | TFDP1 | SUPT5H | SORBS2 |
| BMI1 | EZH2 | LGALS3 | PAK4 | RBP2 | TGFB1I1 | SYK | SOX2 |
| BRAF | FBXO31 | LIMA1 | PBRM1 | RBX1 | TLR3 | TACC3 | SPIN1 |
| BRD7 | FOXM1 | LIMK1 | PCGF2 | RNASEL | TMSB4X | WNT2 | SOX5 |
| BRCA1 | FOS | MAGEA2 | PDCD10 | RPS6KA6 | TNFSF13 | WRN | SP1 |
| BTG3 | FOXO3 | MAGOH | PDIK1L | RSL1D1 | TNFSF15 | WT1 | SPOP |
| C11orf31 | FXR1 | MAD2L1 | PDZD2 | RUNX1 | TOP1 | XAF1 | SRC |
| CAV1 | G6PD | MAGOHB | PDPK1 | RUVBL2 | TP63 | WWP1 | SIRT6 |
| CBX7 | GAPDH | MAP2K1 | PEBP1 | SENP1 | TPR | YAP1 | SIX1 |
| CBX8 | GKN1 | MAP2K3 | PEX19 | SENP2 | TP53 | YPEL3 | SLC13A3 |
| CCND1 | GATA4 | MAP2K2 | PIAS4 | SENP7 | TRIM28 | ZFP36 | SLC16A7 |
| CDK1 | GNG11 | MAP2K6 | PIK3R5 | SERPINE1 | TRPM8 | ZMAT3 | SMARCA4 |
| CDK18 | GLB1 | MAP3K6 | PIK3C2A | SFN | TXN | ZNF148 | SMG1 |
| CDK2AP1 | GRK6 | MAP2K7 | PIM1 | SIK1 | TXNIP | VENTX | WNT16 |
| SGK1 | UBTD1 | USP1 | SIRT1 | SIN3B | TYK2 | VEGFA |  |
