# Supplementary material for: Exploring the Relationship Between Senescence and Colorectal Cancer in Prognosis, Immunity, and Treatment
Source: Front Genet. 2022 Jun 15;13:930248. doi: 10.3389/fgene.2022.930248 (PMC9240351; doi:10.3389/fgene.2022.930248)
Supplement: Supplementary file 3 [file DataSheet1.docx]

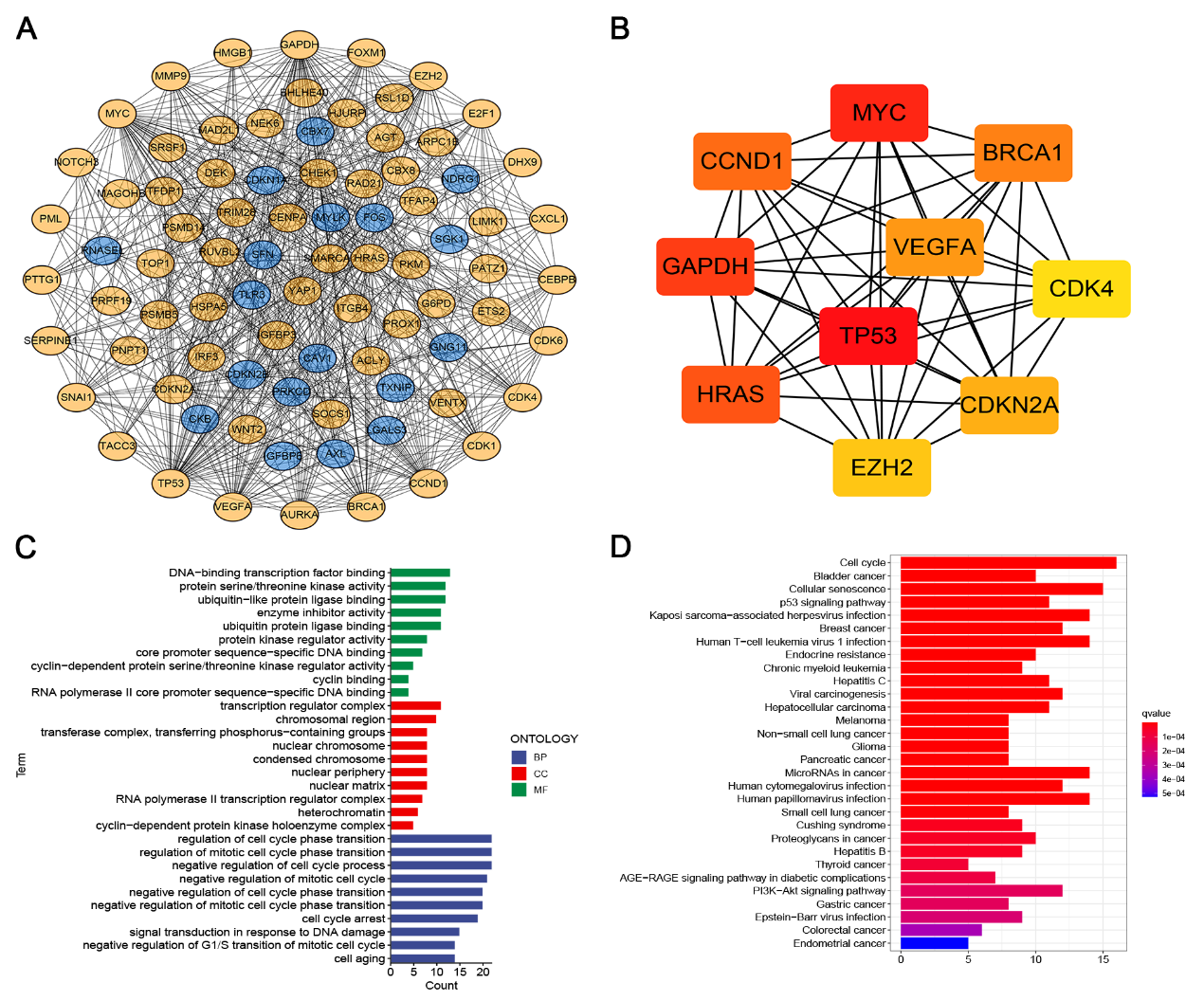


**Supplementary Figure 1** Protein-protein interaction networks and functional enrichment analyses. (A) Protein-Protein interaction analysis was performed to analyze the interaction protein network of 93 DEGs. Orange represents upregulated DEGs and blue represents downregulated DEGs. (B) Top 10 hub genes were identified by cytoHubba plugin. (C-D) The GO (C) and KEGG (D) analysis of 93 DEGs.


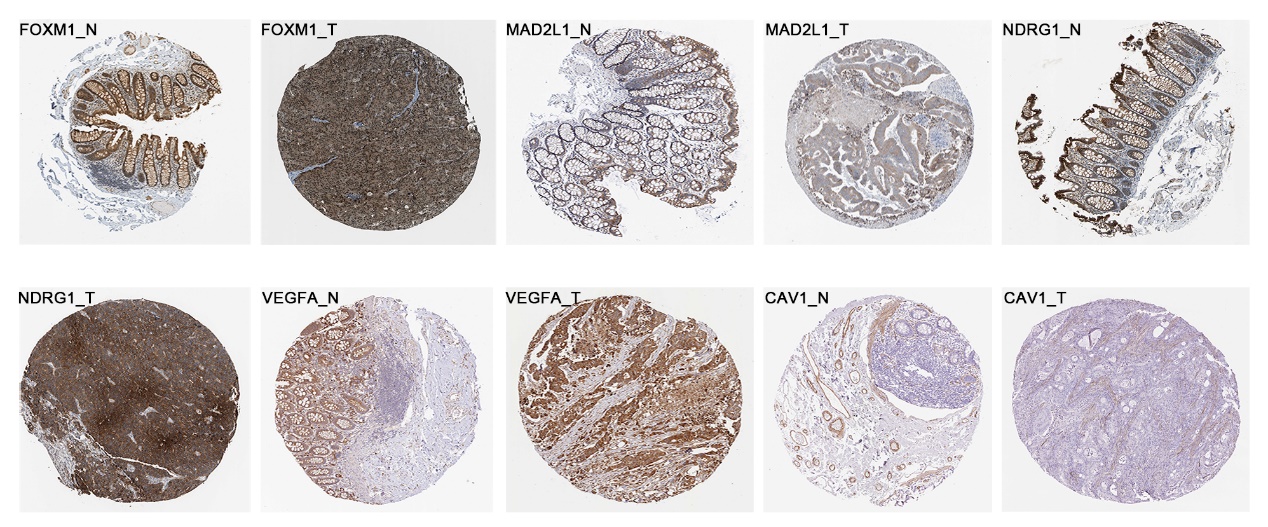


**Supplementary Figure 2** Protein expression of the signature genes in CRC and non-cancerous colorectal tissues via the Human Protein Atlas (HPA) database.


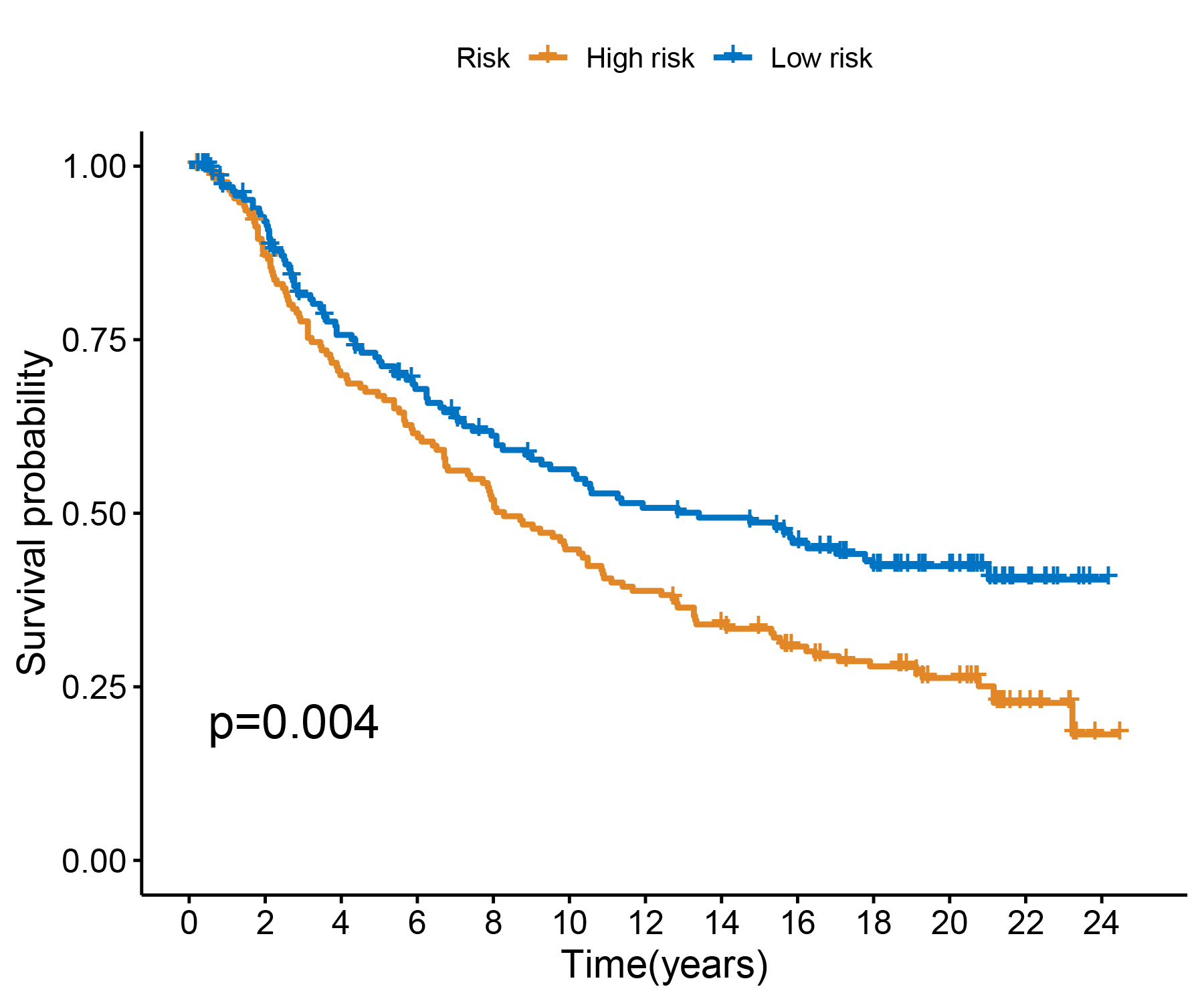


**Supplementary Figure 3** The predictive ability of the signature was also confirmed by in the IMvigor210 cohort.


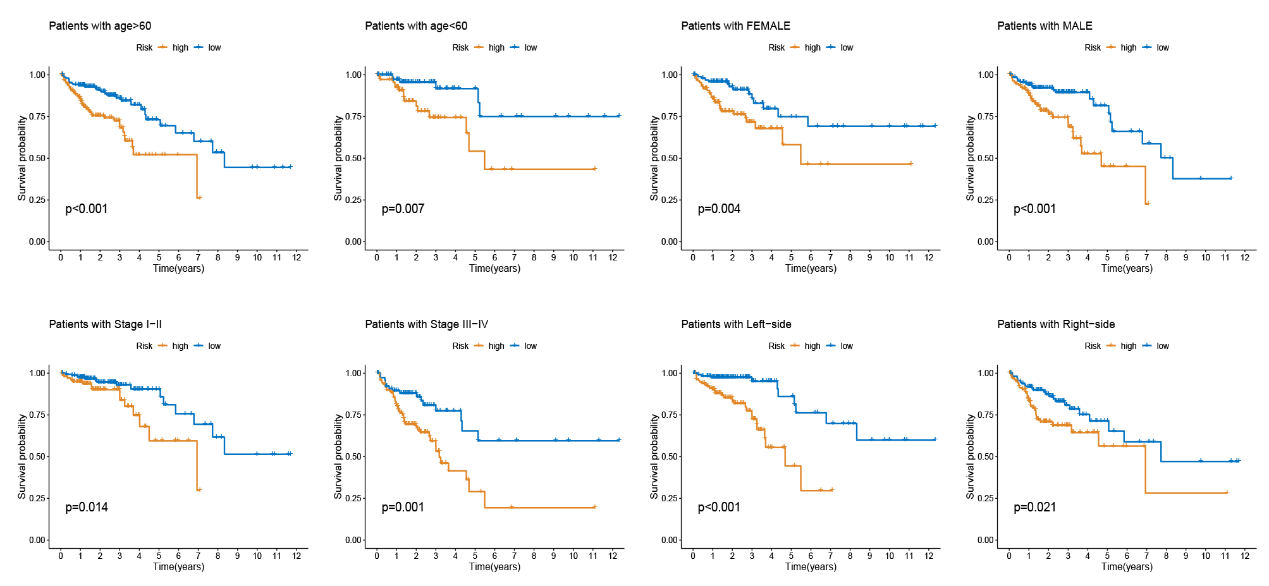


**Supplementary Figure 4** The overall survival rate of patients in two risk groups between distinct clinical variable subgroups.
